# Predictive value of the Essen Stroke Risk Score and Ankle Brachial Index in acute ischaemic stroke patients from 85 German stroke units

**DOI:** 10.1136/jnnp.2008.146092

**Published:** 2008-06-27

**Authors:** C Weimar, M Goertler, J Röther, E B Ringelstein, H Darius, D G Nabavi, In-Ha Kim, Jens Benemann, Hans-Christoph Diener, on behalf of the SCALA Study Group

**Affiliations:** 1 Department of Neurology, University of Duisburg-Essen, Essen, Germany; 2 Department of Neurology, University of Magdeburg, Magdeburg, Germany; 3 Department of Neurology, Klinikum Minden, Minden, Germany; 4 Department of Neurology, University of Muenster, Muenster, Germany; 5 Department of Cardiology, Vivantes Hospital Neukoelln, Berlin, Germany; 6 Department of Neurology, Vivantes Hospital Neukoelln, Berlin, Germany; 7 Medical Department, Sanofi-Aventis, Paris, France

## Abstract

**Background:**

Risk stratification can contribute to individualised optimal secondary prevention in patients with cerebrovascular disease.

**Objective:**

To prospectively investigate the prediction of the Essen Stroke Risk Score (ESRS) and a pathological Ankle Brachial Index (ABI) in consecutive patients hospitalised with acute ischaemic stroke or transient ischaemic attack (TIA) in 85 neurological stroke units throughout Germany.

**Methods:**

852 patients were prospectively documented on standardised case report forms, including assessment of ESRS and ABI. After 17.5 months, recurrent cerebrovascular events, functional outcome or death could be assessed in 729 patients predominantly via central telephone interview.

**Results:**

After discharge from the documenting hospital, recurrent stroke occurred in 41 patients (5.6%) and recurrent TIA in 15 patients (2.1%). 52 patients (7.1%) had died, 33 (4.5%) from cardiovascular causes. Patients with an ESRS ⩾3 (vs <3) had a significantly higher risk of recurrent stroke or cardiovascular death (9.7% vs 5.1%; odds ratio (OR) 2.00, 95% confidence interval (CI) 1.08 to 3.70) and a higher recurrent stroke risk (6.9% vs 3.7%; OR 1.93, 95% CI 0.95 to 3.94). Patients with an ABI ⩽0.9 (vs >0.9) had a significantly higher risk of recurrent stroke or cardiovascular death (10.4% vs 5.5%; OR 2.00, 95% CI 1.12 to 3.56) and a higher recurrent stroke risk (6.6% vs 4.6%; OR 1.47, 95% CI 0.76 to 2.83).

**Conclusion:**

Our prospective follow-up study shows a significantly higher rate of recurrent stroke or cardiovascular death and a clear trend for a higher rate of recurrent stroke in patients with acute cerebrovascular events classified as high risk by an ESRS ⩾3 or a pathological ABI.

Because of the aging population, the incidence of ischaemic stroke (IS) is increasing in industrialised countries with a significant burden from an individual as well as a public health perspective.^1^ In contrast with the incidence of first ever stroke, which is still expected to rise due to an increasing life expectancy,^2^ the rate of recurrent stroke is more susceptible to medical treatment or preventive measures and therefore could be effectively reduced.^3^ ^4^ While predictive models have already proven their usefulness in patients with myocardial infarction and atrial fibrillation, they are still hardly used in treatment decisions following IS or transient ischaemic attack (TIA).

Validated scores exist for the prediction of first stroke,^5^ ^6^ as well as for the prediction of recurrent (cerebro)vascular events.^7^^–^9 Recently, the Essen Stroke Risk Score (ESRS^10^) was derived from the data subset of 6433 cerebrovascular patients in the large scale Clopidogrel versus Aspirin in Patients at Risk of Ischaemic Events (CAPRIE) trial.^11^ On a linear 10 point scale, the ESRS, as presented in table 1, predicts short term (1 year) risk of recurrent stroke. The low risk category (score 0–2) and the higher risk category (score ⩾3) can easily be distinguished. Because the ESRS has been developed and validated only in populations from randomised controlled trials with strict inclusion and exclusion criteria, we performed a prospective validation in the Systemic Risk Score Evaluation in Ischaemic Stroke Patients (SCALA) study on patients with IS and TIA routinely admitted to certified German stroke units.^12^ At baseline, we also assessed the ankle brachial index (ABI) which is an easy to use, inexpensive and reliable tool to identify patients with high atherosclerotic burden and thus high cardiovascular risk. Among trained investigators, test–retest reliability of the ABI is excellent, and a series of large scale epidemiological studies have shown a strong correlation between low ABI scores and (cardiovascular) mortality.^13^ ^14^ Current guidelines of the American Heart Association thus recommend the ABI for screening of asymptomatic patients to identify and treat an increased risk of coronary artery disease and stroke.^15^ Similarly, a strong association was demonstrated between a low ABI and an increased incidence of ischaemic stroke although sensitivity was low.^13^ ^16^^–^18 However, only one study so far has assessed the prognostic value of the ABI in patients with acute cerebrovascular events.^19^ The aims of the present longitudinal study therefore were to validate the prediction of the ESRS with the established cut-off ⩾3 for high risk patients and to investigate the prediction of a pathological ABI for future cerebrovascular events and vascular death in patients after an acute IS or TIA.

**Table 1:** Baseline characteristics on the Essen Stroke Risk Score (ESRS) for patients who were and were not followed-up

Risk factor (points allocated)	With follow-up (n = 729)	Without follow-up (n = 123)
Age 65–75 years (%) (1 point)	35.9	33.3
Age >75 years (%) (2 points)	26.0	36.6*
Arterial hypertension (%) (1 point)	70.4	73.8
Diabetes mellitus (%) (1 point)	26.9	22.8
Previous MI (%) (1 point)	17.1	19.7
Other cardiovascular disease (except MI and atrial fibrillation) (%) (1 point)	36.8	30.1
PAD (%) (1 point)	10.3	10.6
Smoker (%) (1 point)	24.9	23.9
Previous TIA or ischaemic stroke in addition to qualifying event (%) (1 point)	25.4	30.1
Mean ESRS sum score	2.96	3.06

*Significant at p<0.05.

MI, myocardial infarction; PAD, peripheral arterial disease; TIA, transient ischaemic attack.

## METHODS

This prospective observational cohort study (Systemic Risk Score Evaluation in Ischaemic Stroke Patients (SCALA)) was conducted in 85 certified German neurological stroke units, each of which documented 10 consecutive patients with acute IS or TIA on standardised case report forms, during the period from July 2005 to October 2005. Methods and results of baseline data collection have been described previously.^12^ In short, the following exclusion criteria were applied: primary cerebral haemorrhage, intubation and refusal or inability to provide informed consent. Patients were treated according to best current knowledge, and management was not delayed or altered by participation in this study. Patients provided written informed consent for study participation. The study was approved by the ethics committee of the University of Essen and conducted according to the national data protection legislation. The ESRS is a simple sum score calculated as follows: 2 points for age >75 years, each 1 point for age ⩾65–75, arterial hypertension, diabetes mellitus, previous myocardial infarction, other cardiovascular disease (except myocardial infarction and atrial fibrillation), peripheral arterial disease, current or past (<5 years) smoking and previous TIA or ischaemic stroke in addition to qualifying event. The ABI was obtained after a 5 min rest in the supine position from systolic blood pressure readings by Doppler sonography at the ankle (posterior and anterior tibial artery) and at the brachial artery. The highest systolic blood pressure in each leg was then divided by the average systolic pressure in both arms (unless there was a discrepancy of ⩾10 mm Hg between the two arms).

A central follow-up interview via telephone (n = 649) or written questionnaire (n = 80) was performed in 729 participants after 17.5 (SD 0.88) months. No follow-up was available for 123 patients (14.4%) either because they did not consent to follow-up (n = 112) or were reportedly alive but could not be reached (n = 11). Follow-up included screening for recurrent cerebrovascular events and assessment of functional disability scales (Barthel Index, modified Rankin Scale (mRS)) or cause of death. In the case of a recurrent cerebrovascular event or death, confirmation was sought from the family physician, treating hospital or local death registries. Only events after discharge from the documenting hospital were considered.

### Statistics

Categorical variables are presented as percentages and continuous variables as mean (SD) and/or median (quartiles). The χ^2^ test and Fisher’s exact test, as appropriate, were used for comparison of categorical variables. The Wilcoxon rank sum score was used for comparison of non-normally distributed variables. If any variable was not available for all patients, only valid cases were reported. We calculated the time of event free survival by Kaplan-Meier (KM) estimates. To evaluate the performance of the ESRS and ABI, we calculated the area under the curve (AUC) by c statistic and calibration χ^2^ (survival modified Hosmer–Lemeshow). An AUC of 0.5 indicates no discrimination, and an AUC of 1.0 indicates perfect discrimination. Analyses were done with SAS V.8.2 and SPSS V.14.0.2.

## RESULTS

The 85 centres listed in the appendix (available online) consecutively included 852 patients with a mean age of 67.1 (SD 12.4) years and a diagnosis of IS in 82.9% and TIA in 17.1%. Most index events (89.7%) had occurred within the past 7 days prior to study inclusion. Stroke aetiology was classified as large artery disease in 26.0%, small vessel disease in 27.2%, cardioembolic in 23.9% and other or undetermined aetiology in 22.9% of patients. Other baseline characteristics have been reported previously.^12^ Follow-up after 17.5 (SD 0.88) months was possible in 729 patients (85.6%), 17.4% with TIA and 82.6% with IS. Compared with patients who were followed-up, those without follow-up were significantly older (p = 0.043), more often had a pathological ABI (66.7% vs 52.8%; p<0.005) and had more severe baseline stroke severity on the National Institutes of Health-Stroke Scale (mean 6.96 vs. 4.96; p = 0.012) but were not significantly different with regard to their overall ESRS sum score (table 1).

**Figure 1:**
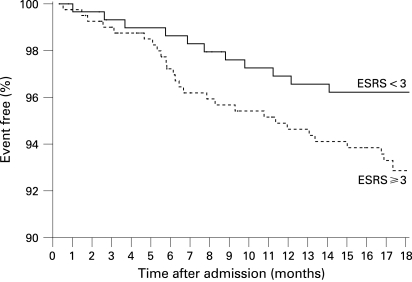
Survival free of recurrent stroke during follow-up in patients with an Essen Stroke Risk Score (ESRS) <3 versus those with a score ⩾3 (n = 700).

**Figure 2:**
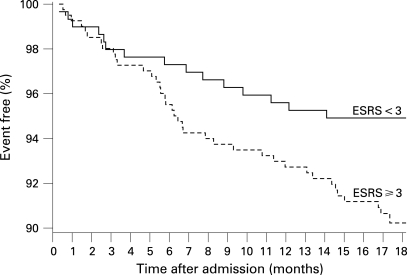
Survival free of recurrent stroke or cardiovascular death during follow-up in patients with an Essen Stroke Risk Score (ESRS) <3 versus those with a score ⩾3 (n = 700).

A recurrent fatal or non-fatal stroke was reported by or in 41 patients (5.6%) and a recurrent TIA by 15 patients (2.1%). Confirmation of these events by the family practitioner or treating hospital was obtained in 37 and 11 patients, respectively. One event occurred during carotid endarterectomy which, together with endovascular stenting, was performed in 32 patients. Recurrent stroke or cardiovascular death occurred in 60 patients. Overall, 52 patients (7.1%) died during follow-up (seven because of the initial stroke, 12 because of a recurrent stroke, five because of myocardial infarction, nine because of other cardiovascular events, 13 because of other causes and six as a result of an unknown cause). Of 677 surviving patients, 179 (26.4%) had not regained functional independence (mRS >2), 85 patients (12.6%) were largely independent (mRS 2), 398 patients (58.8%) reported no or only minor disability (mRS <2) and no information on functional outcome was available in 15 patients (2.2%). Surviving patients with a recurrent stroke had a significantly worse functional status on follow-up (median mRS 4) compared with event free patients (median mRS 1). No antithrombotic medication at follow-up was reported by 44 patients (6.5%). A total of 287 patients (42.4%) were receiving aspirin, 148 (21.9%) phenprocoumon or warfarin (seven with additional aspirin), 107 (15.8%) clopidogrel (12 with additional aspirin), 52 (7.7%) aspirin/dipyridamol, two (0.3%) heparin and eight (1.2%) various study medications (medication not further specified in 29 patients). Complete information for calculation of the ESRS was available in 700 patients and for the ABI in 692 patients. Recurrent stroke occurred in 11 (3.7%) of 296 patients with ESRS <3 (or 17/5.7% including TIA) compared with 28 (6.9%) (or 35/8.7% including TIA) of 404 patients with ESRS ⩾3 (odds ratio (OR) for stroke 1.93, 95% confidence interval (CI) 0.95 to 3.94). The survival proportion free of recurrent stroke stratified by the ESRS is shown in fig 1. The AUC assessed by c statistics was 0.56 (NS). The risk of the combined vascular endpoint recurrent stroke or cardiovascular death was significantly higher in patients with ESRS ⩾3 (39 events/9.7%) compared with patients with ESRS <3 (15 events/5.1%; OR 2.00, 95% CI 1.08 to 3.70; p = 0.031). Stratified KM estimates are shown in fig 2. The AUC assessed by c statistics was 0.61 (95% CI 0.54 to 0.69; p = 0.006).

**Figure 3:**
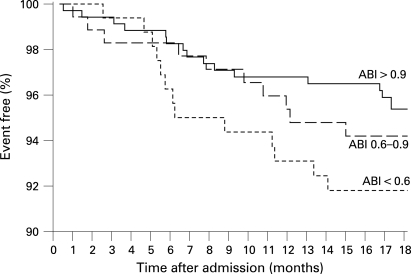
Survival free of recurrent stroke during follow-up in patients with an Ankle Brachial Index (ABI) >0.9 versus those with an index of 0.6–0.9 and <0.6 (n = 692).

**Figure 4:**
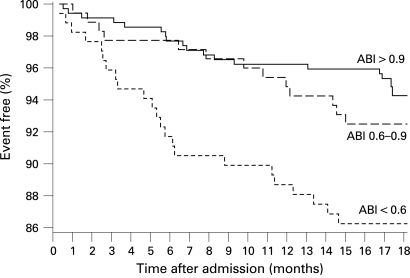
Survival free of recurrent stroke or cardiovascular death during follow-up in patients with an Ankle Brachial Index (ABI) >0.9 versus those with an index of 0.6–0.9 and <0.6 (n = 692).

Recurrent stroke occurred in 16 (4.6%) of 346 patients with an ABI >0.9 (or 20/5.8% including TIA) compared with 23 (6.6%) (or 32/9.2% including TIA) of 346 patients with an ABI ⩽0.9 (OR for stroke 1.47, 95% CI 0.76 to 2.83) which was mainly due to the high stroke risk of 7.6% in 170 patients with an ABI <0.6. The survival proportion free of recurrent stroke stratified by ABI is shown in fig 3. The AUC assessed by c statistics was 0.56 (NS). The risk of the combined vascular end point was significantly higher in patients with ABI ⩽0.9 (36 events/10.4%) compared with patients with ABI >0.9 (19 events/5.5%; OR 2.00, 95% CI 1.12 to 3.56; p = 0.024). Stratified KM estimates are shown in fig 4. The AUC assessed by c statistics was 0.61 (95% CI 0.53 to 0.69; p = 0.006). The correlation between the ESRS and ABI in patients with follow-up was low (r = 0.166, p<0.001). The combination of a high risk on both ESRS and ABI did not result in an improved risk prediction for stroke (6.7% vs 5.2%; OR 1.31, 95% CI 0.67 to 2.54; p = 0.482) or for the combined vascular end point (10.8% vs 6.3%; OR 1.79, 95% CI 1.02 to 3.15; p = 0.048).

**Figure 5:**
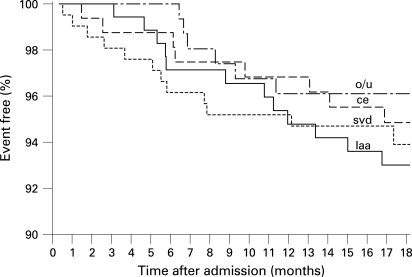
Survival free of recurrent stroke during follow-up stratified by aetiology (large artery atherosclerosis (laa, n = 178), cardiac embolism (ce, n = 163), small vessel disease (svd, n = 209) and other/undetermined (o/u, n = 160).

No significant differences or relevant trends in the risk of recurrent stroke were found for different stroke aetiologies according to the TOAST classification (fig 5).

## DISCUSSION

In this study, we evaluated the ESRS and ABI for identification of patients at high risk of stroke or cardiovascular death after a preceding cerebrovascular ischaemic event. Only a few prognostic instruments for identification of cerebrovascular patients at high risk have been prospectively validated to date and are rarely used in clinical routine. We prospectively assessed the ESRS and ABI in consecutive patients with acute TIA or IS admitted to a large number of acute stroke units covering all geographic areas in Germany. Patients were included consecutively provided they could give informed consent, representing approximately 80–90% of unselected patients admitted to German stroke units. Thus with the exception of severely aphasic and severely ill patients, the population in our study can be regarded as representative of acute stroke units. While both scoring instruments (ABI and ESRS) are simple to apply, their combination did not improve overall prediction, which may be due to their low correlation (Pearson correlation coefficient 0.21)^12^ or the low event rates during follow-up, resulting in a wide CI. Similarly, stratification by type of stroke according to the TOAST criteria in our study did not show any clear trend in risk of recurrent stroke and therefore would not add any predictive accuracy.

Our study on ESRS and ABI in cerebrovascular patients has three major limitations: we did not assess and therefore were unable to consider recurrent cerebrovascular events or cardiovascular death during the acute hospital stay, resulting in lower event rates than expected from other hospital based studies. Because of the low number of stroke events during follow-up, we failed to demonstrate statistically significant differences between high risk and low risk patients for the end point of recurrent stroke, although clear trends for higher stroke recurrence were seen in patients with ESRS ⩾3 or ABI <0.6. Statistically significant differences were found for the combined vascular end point with higher event rates in patients with ESRS ⩾3 or ABI ⩽0.9. Unfortunately, the number of end point events was insufficient to provide meaningful risk stratifications of smaller ESRS or ABI categories and confidence intervals remain wide for the KM estimates which can explain the delayed segregation of the KM curves. A higher follow-up percentage than 85.6% would have been unlikely to change our results because most patients without follow-up simply did not provide informed consent for follow-up and citizen registries were consulted before any patients was considered lost.

Furthermore, the rates of recurrent stroke in the high and low risk strata of the ESRS were very similar to the CAPRIE data set initially used for model development^10^ and the ESPS-2 data used for its retrospective validation^20^ and therefore confirm its predictive value in consecutive patients treated with modern prevention strategies in acute stroke units. Both retrospective analyses of CAPRIE and ESPS2 could also show a steady increase in the risk of stroke with increasing ESRS sum score and an amplified (although non-significant) benefit of clopidogrel or aspirin plus dipyridamole over aspirin in patients with ESRS ⩾3. Secondly, atrial fibrillation was not investigated as an independent predictor or included on development of the ESRS. However, atrial fibrillation has not been identified as an independent risk factor in other follow-up studies either,^7^ ^8^ and the risk of stroke recurrence in patients with a cardioembolic stroke aetiology (most of whom had atrial fibrillation) was not significantly different from other aetiologies. On the other hand, we did not exclude patients with cardioembolic stroke aetiology. Although exclusion of patients with non-atherothrombotic stroke might result in a better prediction of the ESRS and ABI, we aimed to demonstrate the general applicability of the two instruments without additional diagnostic work-up to exclude cardioembolic aetiologies.

Finally, the prediction of the ESRS was based solely on clinical variables, while the ABI assesses generalised atherosclerosis only, which is responsible for less than half of all strokes. In comparison, another clinical scoring system, developed by Hankey *et al* predicting various vascular events (stroke, coronary events, vascular death) at 1 and 5 years later found an AUC value of 0.65 on external validation in the UK–TIA cohort.^21^ Likewise, the SPI-II found an AUC of 0.63 for prediction of stroke or death within 2 years in independent research populations.^7^ Both scores therefore have comparable predictive accuracy compared with our scores for the combined endpoint recurrent stroke/cardiovascular death. Neither one of these scales however has been prospectively validated for prediction of stroke in a non-research population. We could not compare the predictive accuracy between these scales and the ESRS in our study population because not all variables from the other scales had been prospectively documented and the number of outcome events would have been too small to detect any statistically significant differences. As previously reported, an important finding in our study was the high prevalence of pathological ABI values in more than half of all patients, which can be attributed to the inclusion of consecutive patients with acute ischaemic events as well as to a more comprehensive definition of pathological ABI values (⩽0.9 vs >0.9) in our trial.^12^ Although prediction of stroke could be improved by dichotomising the ABI at 0.6, all patients with a low ABI should be considered at high risk for any cardiovascular event, including death.^19^

In conclusion, the ESRS is convenient to use, targets a distinctly important clinical outcome and is reasonably accurate for clinical stratification of high risk patients. Both the ESRS and ABI seem suitable for routine application to increase awareness of recurrent stroke risk in cerebrovascular patients. Whether patients at high risk according to the ABI or ESRS benefit from intensified medical prevention strategies is difficult to assess because of the high number of end points needed. Because of its potential for optimising secondary prevention strategies, this question is of major relevance to public health decisions and should be assessed in future secondary prevention trials. In addition, high risk patients may constitute the ideal target population for clinical trials of more aggressive medical prevention strategies which may also imply a higher associated risk. Moreover, by including only patients at higher risk of recurrent stroke, future trials could achieve the necessary number of endpoint events with fewer patients or within shorter follow-up periods.
